# Supplementary material for: A brain microvasculature endothelial cell‐specific viral vector with the potential to treat neurovascular and neurological diseases
Source: EMBO Mol Med. 2016 Apr 22;8(6):609–25. doi: 10.15252/emmm.201506078 (PMC4888852; doi:10.15252/emmm.201506078)
Supplement: Supplementary file 1 — Appendix [file EMMM-8-609-s001.pdf]

APPENDIX - A brain microvasculature endothelial cell-specific viral vector with the potential to treat neurovascular and neurological diseases

Jakob Körbelin, Godwin Dogbevia, Stefan Michelfelder, Dirk A. Ridder, Agnes Hunger, Jan Wenzel, Henning Seismann, Melanie Lampe, Jacqueline Bannach, Manolis Pasparakis, Jürgen A. Kleinschmidt, Markus Schwaninger, Martin Trepel

Table of content

Appendix Figure S1 - *In vivo* bioluminescence images of mice after tail vein injection of recombinant AAV2 vectors displaying the brain-targeting peptide NRGTEWD (BR1) 2

Appendix Figure S2 - AAV-BR1-mediated long-term luminescence in the brain with exact graphic delineation of the region of interest (ROI) 3

Appendix Figure S3 - Quantification of vector-transduced endothelial cells in different areas of the CNS based on transgene-mediated fluorescence 4

Appendix Figure S4 – String vessels formed in Nemo^{beko} mice 5

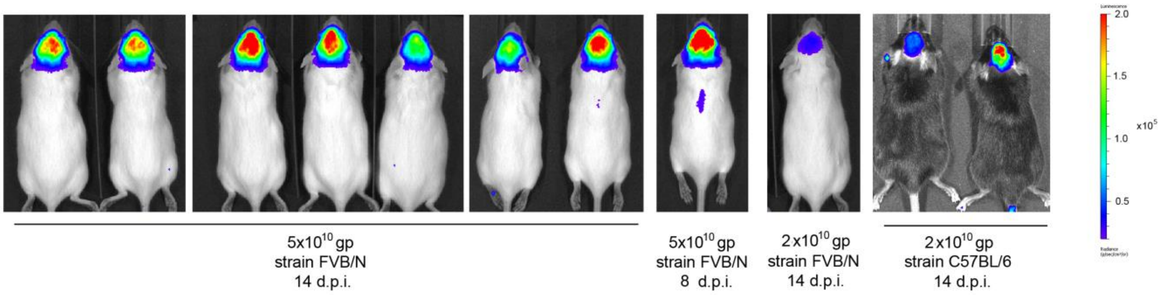

Appendix Figure S1 - *In vivo* bioluminescence images of mice after tail vein injection of recombinant AAV2 vectors displaying the brain-targeting peptide NRGTEWD (BR1)
Mice of different strains (FVB/N and C57BL/6) were treated in different settings ($2x10^{10}$ - $5x10^{10}$ genomic particles/mouse, age 8-12 weeks) with vectors harboring the luciferase gene under control of the CAG promoter. Images were taken at different time points (d.p.i. = days post injection).

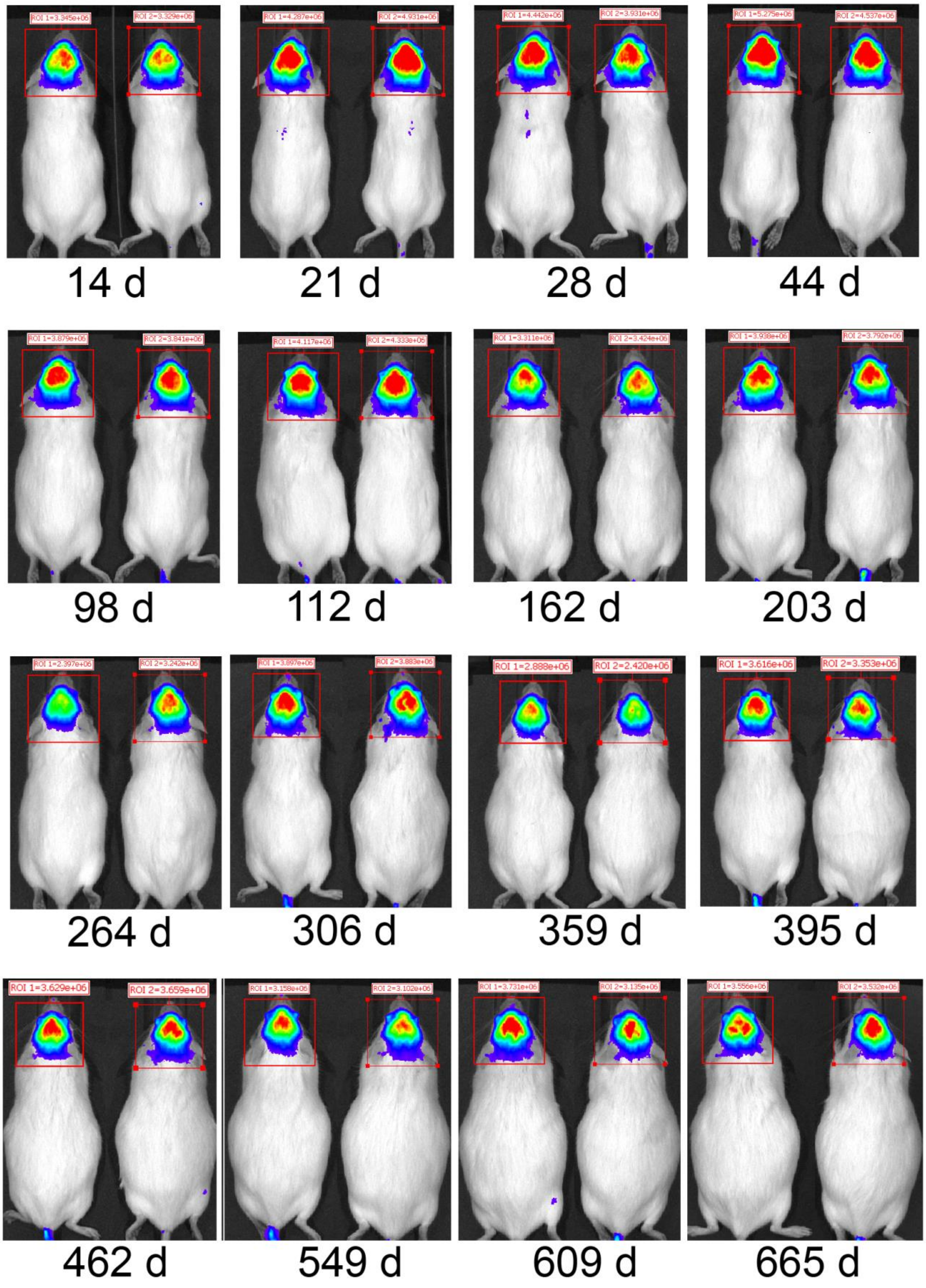

Appendix Figure S2 - AAV-BR1-mediated long-term luminescence in the brain with exact graphic delineation of the region of interest (ROI)

AAV-BR1 luciferase vector under control of the CAG promoter was administered intravenously ($5x10^{10}$ genomic particles/mouse, age 8 weeks). Long-term transgene expression was quantified in the indicated ROI (highlighted as red squares) by bioluminescence imaging at 16 time points during a 665 days period (n = 2 animals)

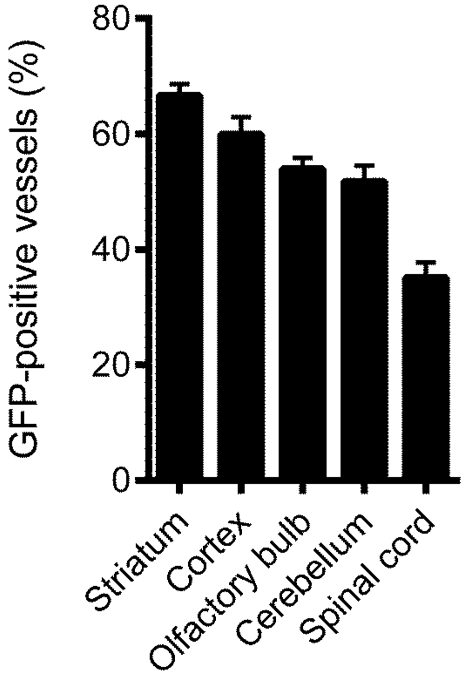

Significant differences:

Spinal cord vs. Striatum: p < 0.0001
Spinal cord vs. Cortex:p < 0.0001
Spinal cord vs. Olfactory bulb: p < 0.0001
Spinal Cord vs. Cerebellum: p = 0.0005
Striatum vs. Olfactory bulb: p = 0.0005
Striatum vs. Cerebellum: p = 0.0038

Appendix Figure S3 - Quantification of vector-transduced endothelial cells in different areas of the CNS based on transgene-mediated fluorescence
GFP-positive vessels were counted as percentage of CD31-positive vessels 14 days after i.v. injection of AAV-BR1-CAG-eGFP (1.8×10^{11} genomic particles/mouse, age 8 weeks). Data were analyzed by one-way ANOVA, followed by Turkey's multiple comparison test. Data are shown as mean +SEM (n = 6 mice/group). The individual adjusted p values are indicated on the right.

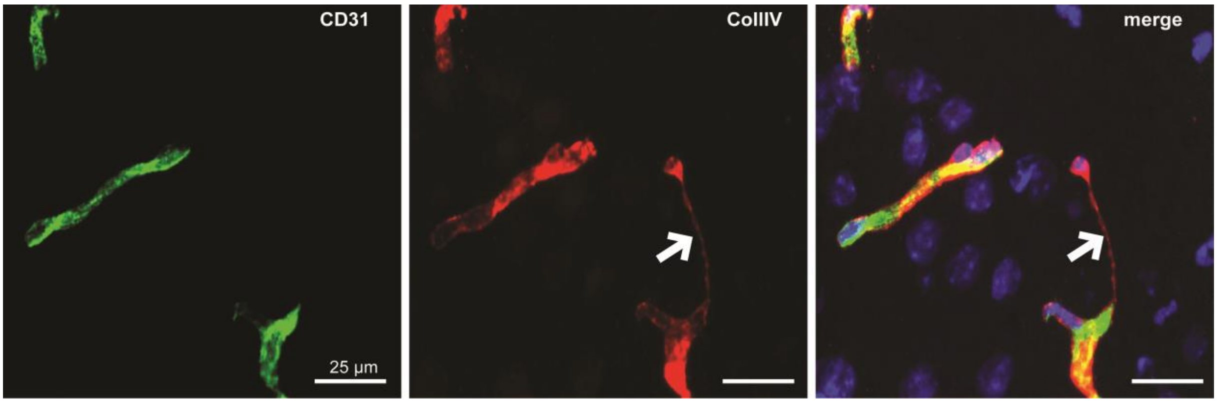

Appendix Figure S4 – String vessels formed in Nemo*beko* mice

Representative high magnification immunostainings of string vessels. The endothelial cell marker CD31 (green) and collagen IV (red) as an integral basement membrane component showing empty basement membrane strands (white arrow) known as string vessels. Scale bars represent 25µm.
