# Supplementary material for: A brain microvasculature endothelial cell‐specific viral vector with the potential to treat neurovascular and neurological diseases
Source: EMBO Mol Med. 2016 Apr 22;8(6):609–25. doi: 10.15252/emmm.201506078 (PMC4888852; doi:10.15252/emmm.201506078)
Supplement: Supplementary file 2 — Expanded View Figures PDF [file EMMM-8-609-s002.pdf]

Expanded View Figures

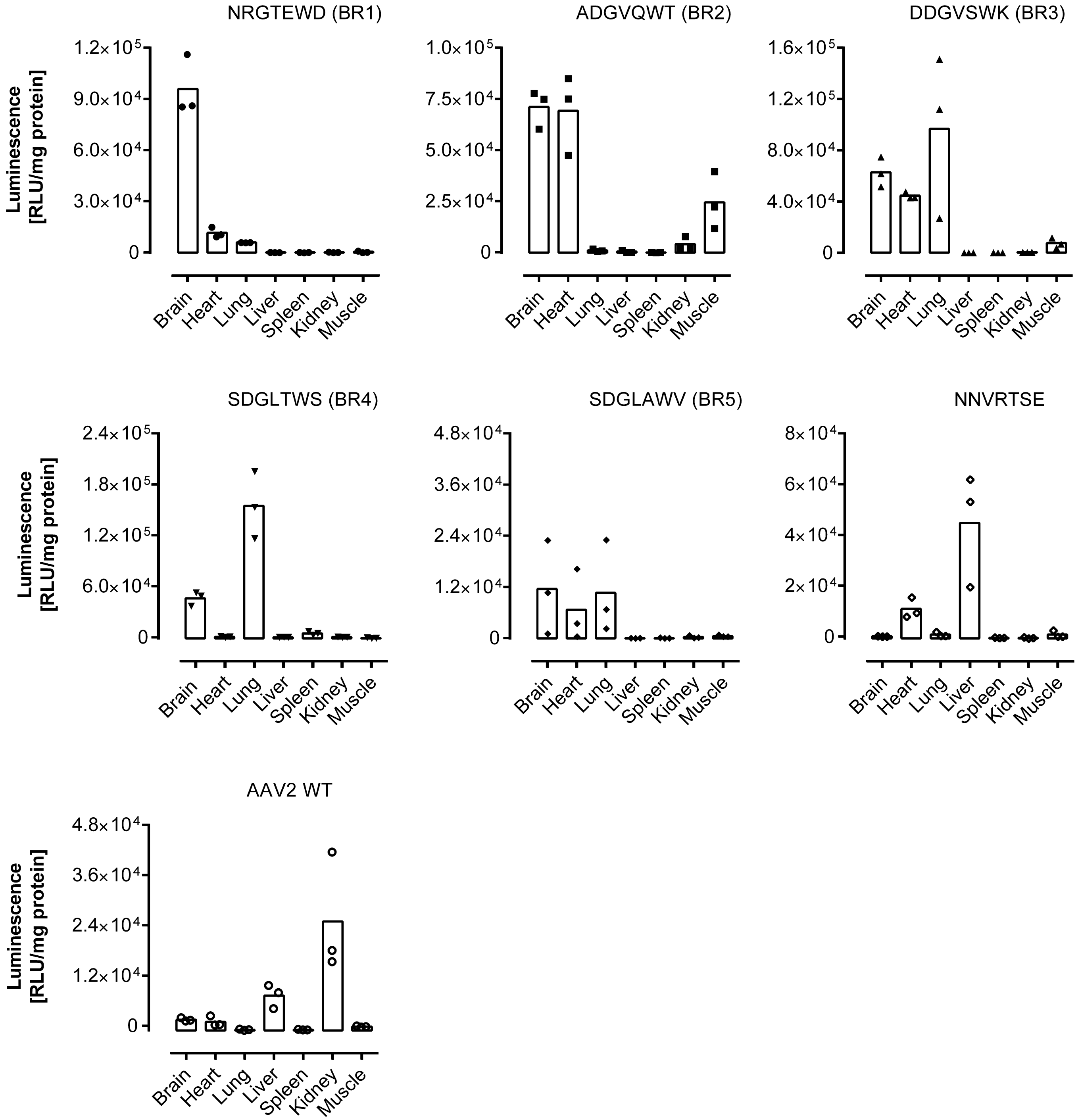

Figure EV1. Luminescence mediated by recombinant AAV2 vectors displaying brain-targeted peptides that were enriched by *in vivo* screening of a random AAV display peptide library.

Tissues were analyzed 28 days after tail vein injection of vectors harboring the luciferase gene under the control of the CMV promoter (5×10^{10} genomic particles/mouse, age 8–12 weeks). Luminescence was normalized to total protein content in the tissue lysates. Data are shown as bars (mean) with plotted individual data points ($n = 3$ animals/group).

 © 2016 The Authors

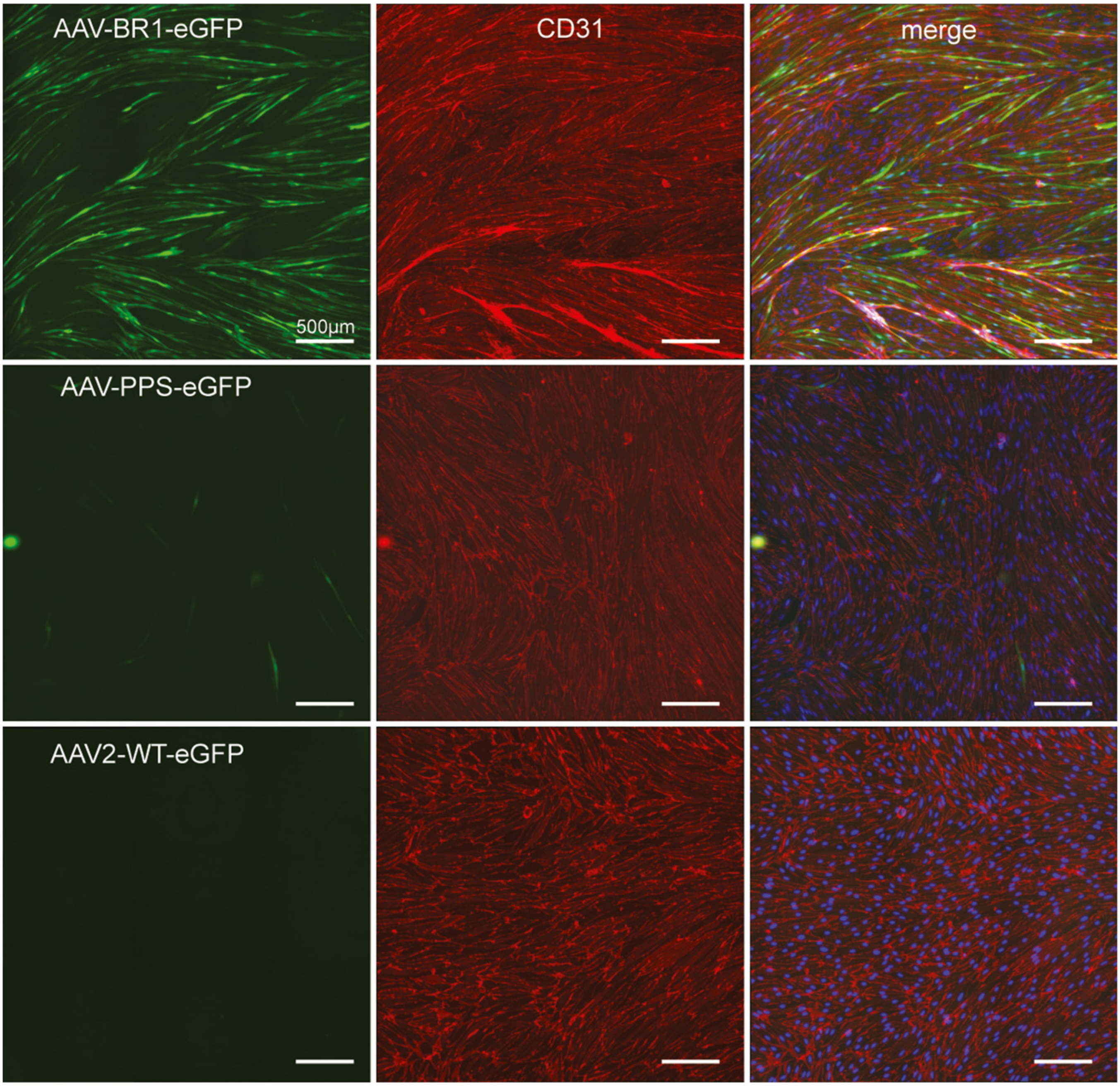

Figure EV2. Mouse primary brain endothelial cells transduced by brain-targeted vectors.
Vector-mediated eGFP expression under the control of the CAG promoter (green), CD31 staining (red), and DAPI (blue; only right panel), 10 days after infection with 1×10^{10} genomic particles of recombinant vector per well. AAV-BR1-eGFP (upper panel), AAV-PPS-eGFP (middle panel), and AAV2-WT-eGFP (lower panel).

© 2016 The Authors

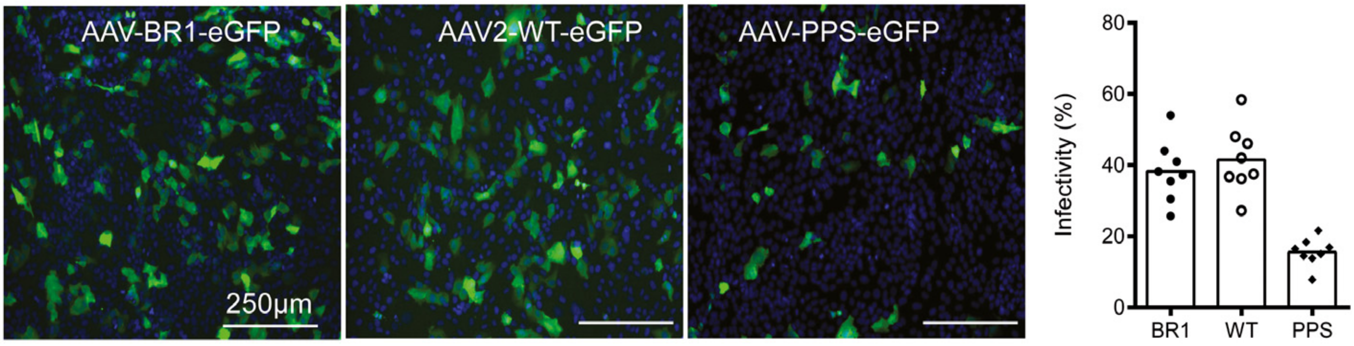

Figure EV3. Immortalized human cerebral microvascular endothelial cells (hCMEC/D3) transduced by brain-targeted vectors.
Vector-mediated eGFP expression under the control of the CAG promoter (green) and DAPI staining of nuclei (blue) are shown. AAV-BR1-eGFP (left panel), AAV2-WT-eGFP (middle panel), and AAV-PPS-eGFP (right panel), 4 days after infection with 1.6×10^{10} genomic particles of recombinant vector per well. The bar graph on the right shows infectivity, counted as GFP-positive in eight randomly taken images from two wells per virus. Data are shown as bars (mean) with plotted individual data points.

 © 2016 The Authors

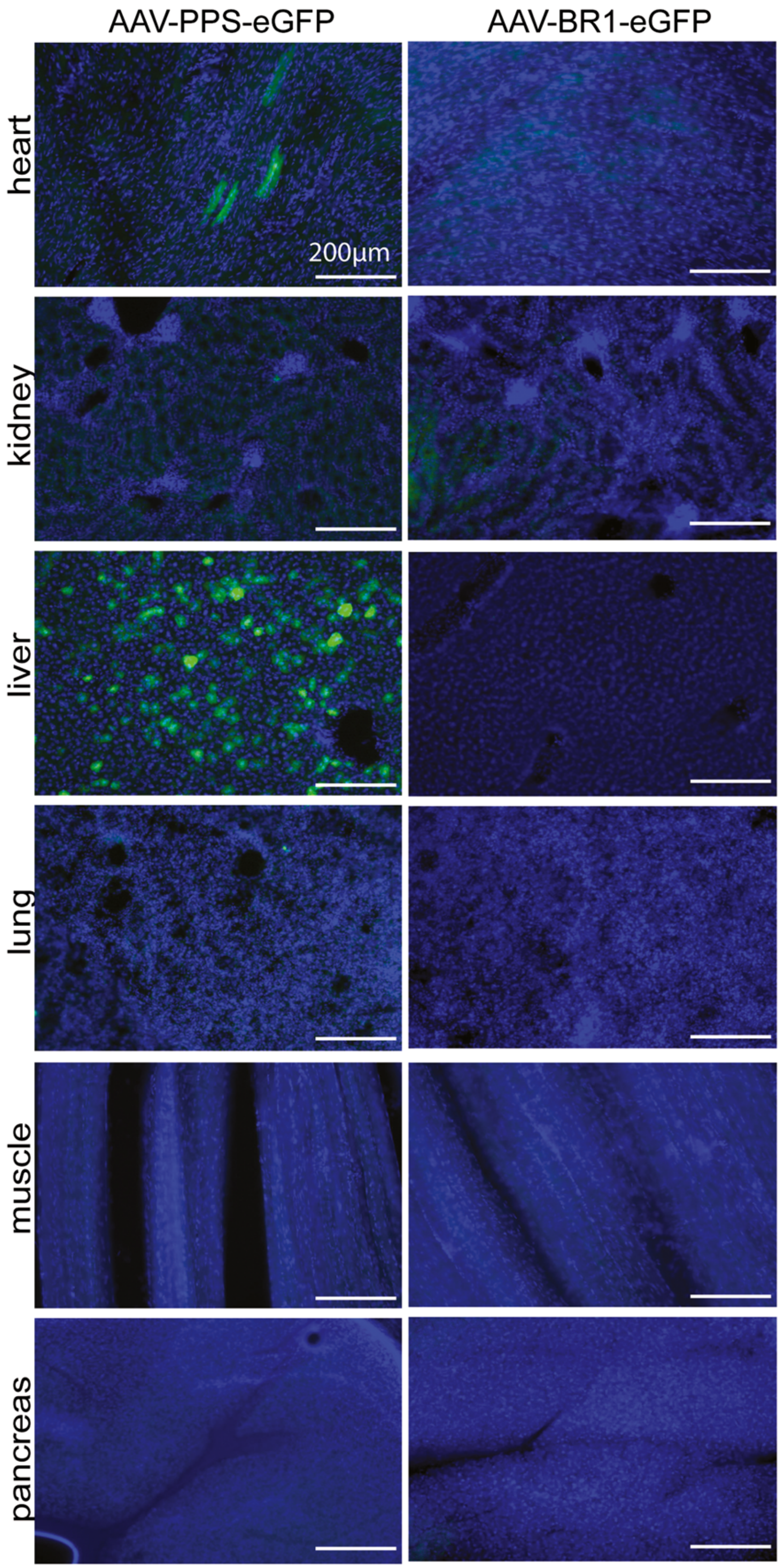

Figure EV4. Off-target transgene expression in mice injected with eGFP vectors.
Tissue sections of different organs other than the brain analyzed 14 days after intravenous injection of 1.8×10^{11} genomic particles/mouse, age 8 weeks. Tissues were analyzed for vector-mediated eGFP expression under the control of the CAG promoter (green) and DAPI staining (blue). AAV-PPS-eGFP (left panel) or AAV-BR1-eGFP (right panel). Scale bars represent 200 µm.

© 2016 The Authors

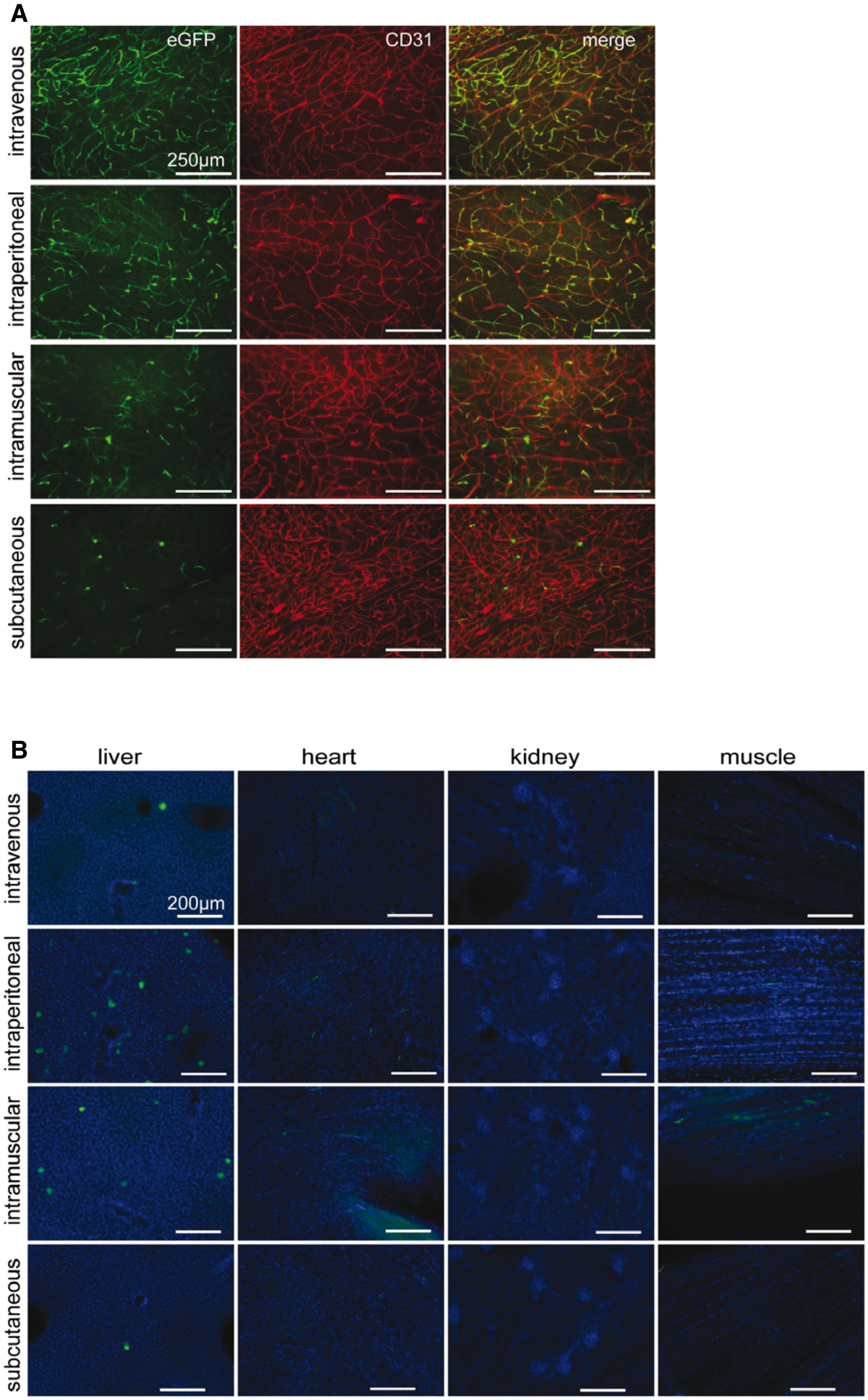

Figure EV5. Effects of different injection routes on AAV-BR1 targeting.

A Representative images of mouse brain cortices showing the extent of endothelial transduction by AAV-BR1-CAG-eGFP injected through 4 different routes (intravenous: i.v., intraperitoneal: i.p., intramuscular: i.m., and subcutaneous: s.c.) 14 days after virus injection. Scale bars represent 250 μm.

B Tissue sections of different organs (liver, heart, kidney, and muscle) analyzed 14 days after injection of 1.8×10^{11} genomic particles through 4 different routes (as in A). Tissues were analyzed for vector-mediated eGFP expression (green) and DAPI staining (blue). Scale bars represent 200 μm.

 © 2016 The Authors
