# Supplementary figures and images for: A brain microvasculature endothelial cell‐specific viral vector with the potential to treat neurovascular and neurological diseases
Source: EMBO Mol Med. 2016 Apr 22;8(6):609–25. doi: 10.15252/emmm.201506078 (PMC4888852; doi:10.15252/emmm.201506078)

Original Gel\_ figure 7

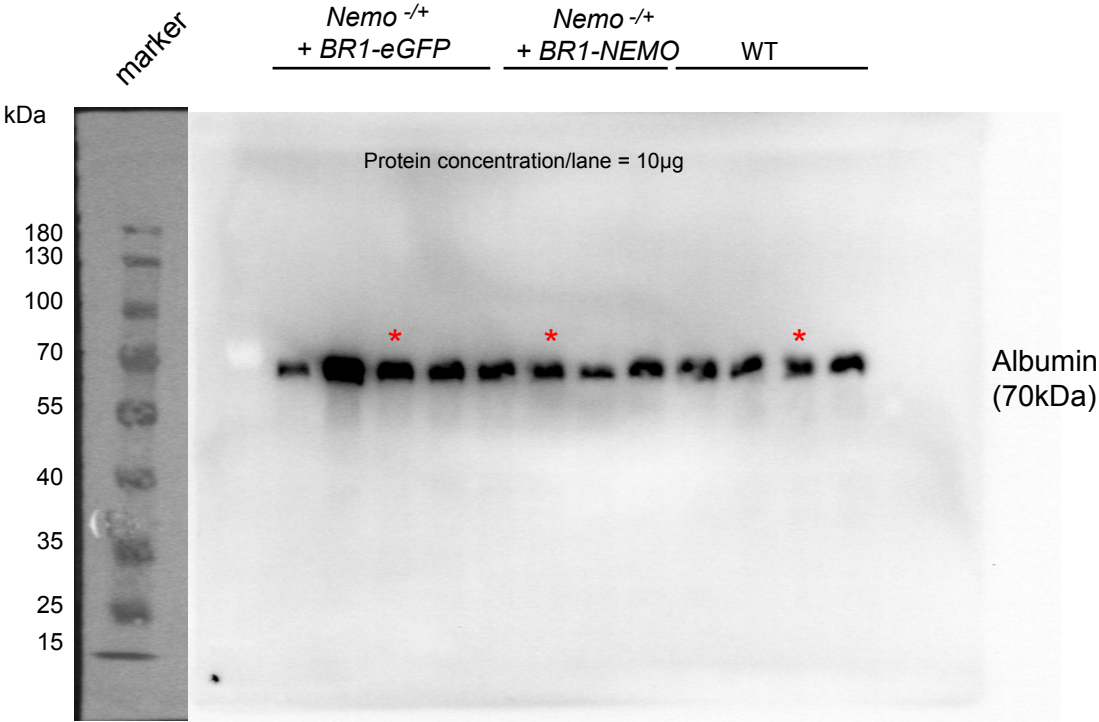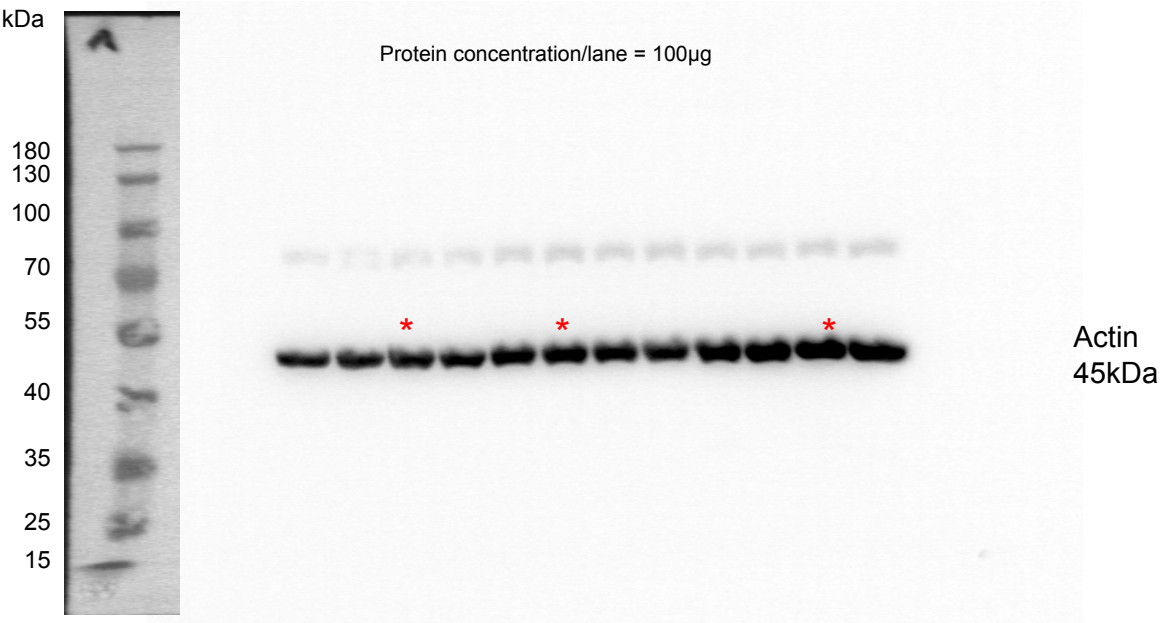

Supplement: Supplementary file 4 — Source Data for Figure 7 [file EMMM-8-609-s003.pdf]

## Original Gel\_figure 8

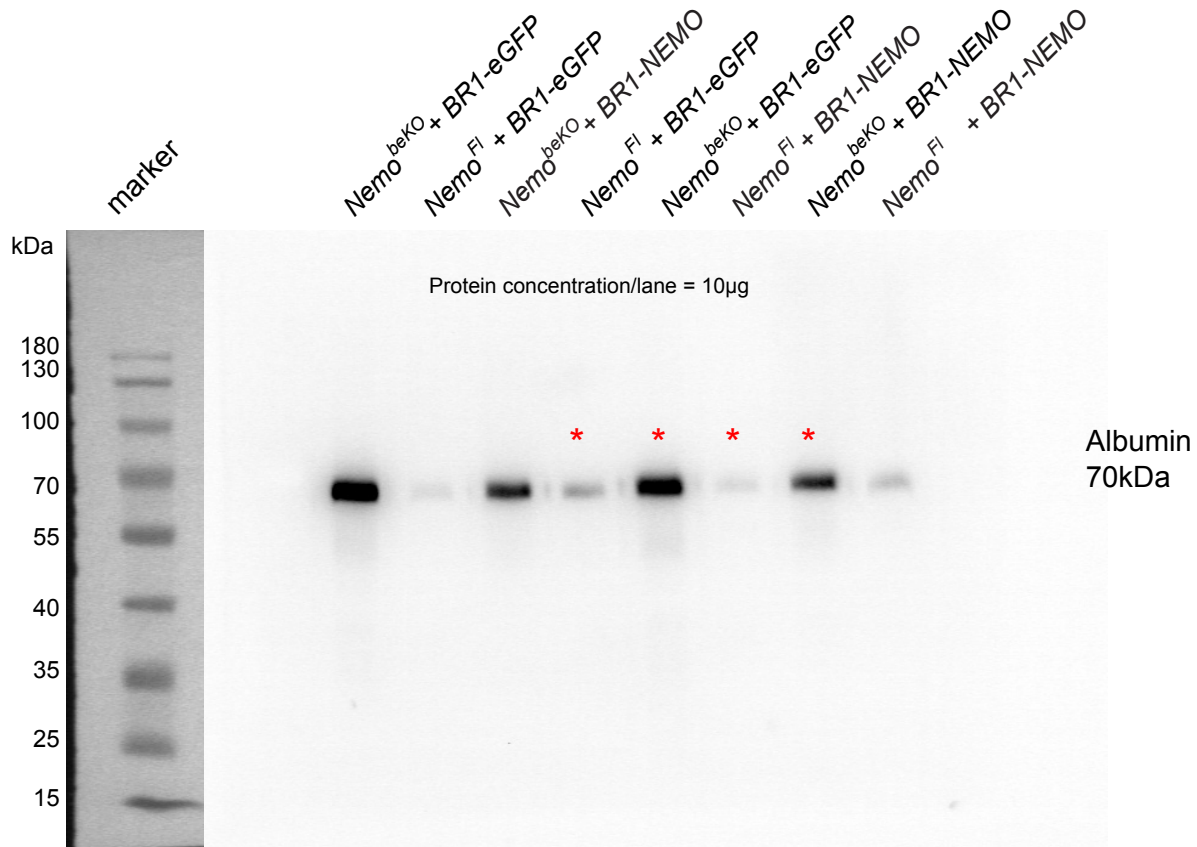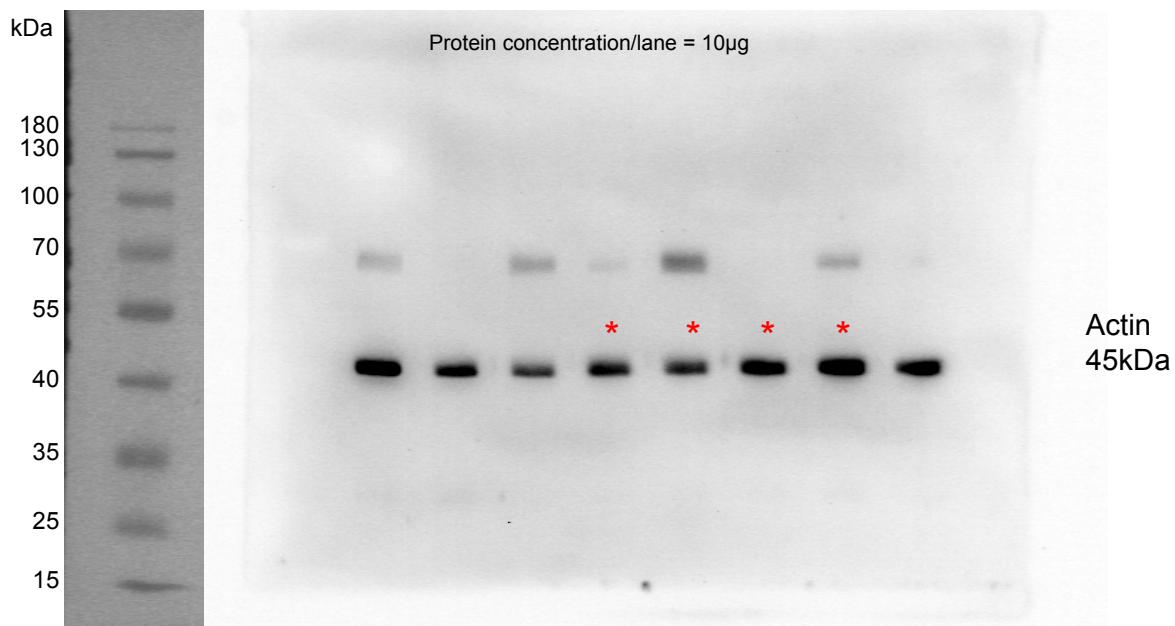

# Original Gel\_figure 8

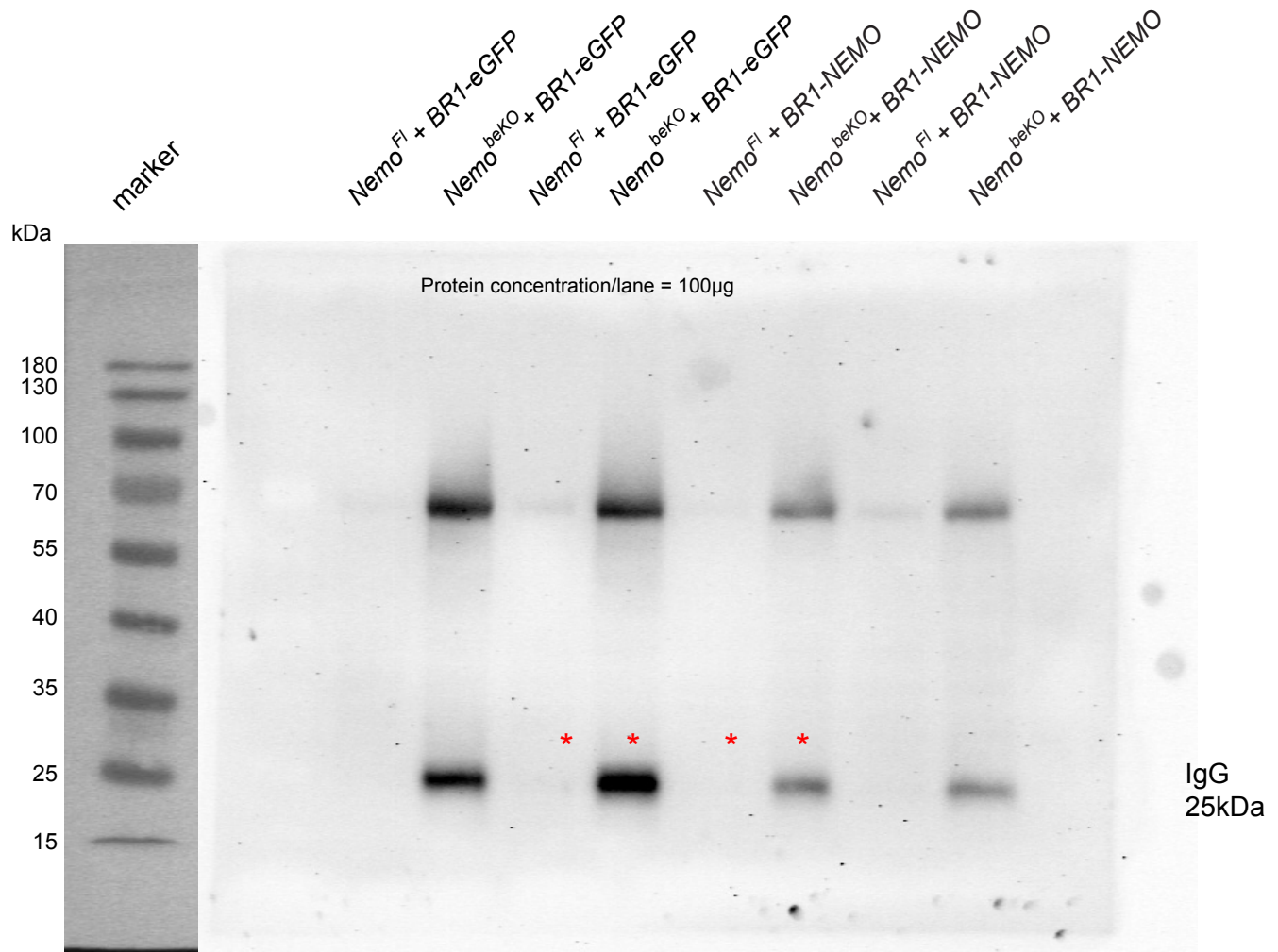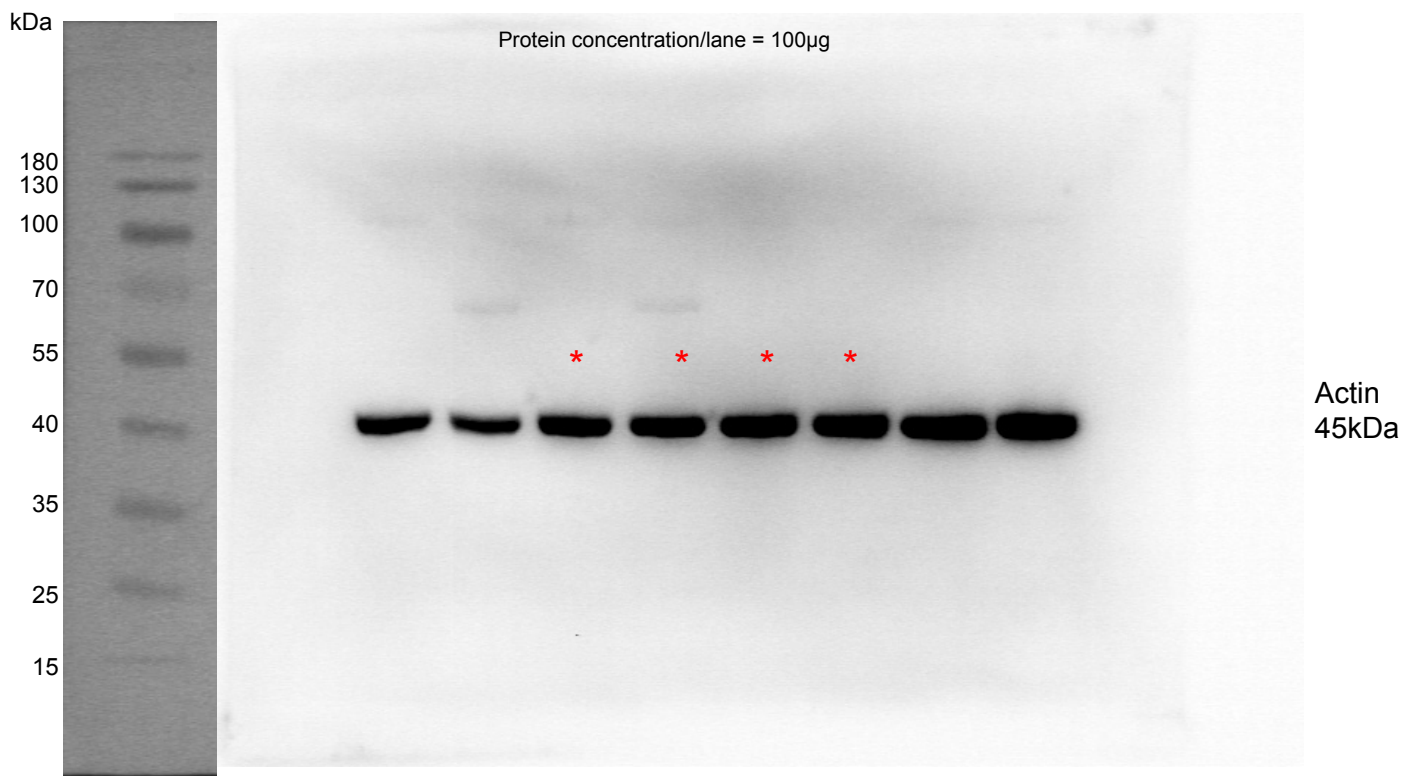

Supplement: Supplementary file 5 — Source Data for Figure 8 [file EMMM-8-609-s004.pdf]
